# Recovery Potential in Patients After Cardiac Arrest Who Die After Limitations or Withdrawal of Life Support

**DOI:** 10.1001/jamanetworkopen.2025.1714

**Published:** 2025-03-25

**Authors:** Jonathan Elmer, Patrick J. Coppler, Cecelia Ratay, Alexis Steinberg, Sara DiFiore-Sprouse, Nicholas Case, Baruch Fischhoff, Maria De-Arteaga, Alain Cariou, Alejandro A. Rabinstein, Andrea O. Rossetti, Ankur A. Doshi, Bradley J. Molyneaux, Cameron Dezfulian, Carolina B. Maciel, Christoph Leithner, Cindy H. Hsu, Claudio Sandroni, David M. Greer, David B. Seder, Francis X. Guyette, Fabio Silvio Taccone, Hiromichi Naito, Jasmeet Soar, Jean-Baptiste Lascarrou, Jerry P. Nolan, Karen G. Hirsch, Katherine M. Berg, Marion Moseby-Knappe, Markus B. Skrifvars, Michael C. Kurz, Min Jung Kathy Chae, Mypinder S. Sekhon, Nicholas J. Johnson, Pedro Kurtz, Romergryko G. Geocadin, Sachin Agarwal, Teresa L. May, Theresa M. Olasveengen, Clifton W. Callaway

**Affiliations:** 1 Department of Emergency Medicine, University of Pittsburgh School of Medicine, Pittsburgh, Pennsylvania; 2 Department of Critical Care Medicine University of Pittsburgh School of Medicine, Pittsburgh, Pennsylvania; 3 Department of Neurology, University of Pittsburgh School of Medicine, Pittsburgh, Pennsylvania; 4 Department of Engineering and Public Policy, Carnegie Mellon University, Pittsburgh, Pennsylvania; 5 Department of Information, Risk and Operations Management, McCombs School of Business, University of Texas at Austin; 6 Médecine Intensive et Réanimation–Hôpital Cochin, Assistance Publique Hôpitaux de Paris Centre–Université Paris Cité, Paris, France; 7 Department of Neurology, Mayo Clinic, Rochester, Minnesota; 8 University Hospital and University of Lausanne, Lausanne, Switzerland; 9 Department of Neurology, Brigham and Women’s Hospital, Boston, Massachusetts; 10 Baylor College of Medicine, Houston, Texas; 11 Departments of Neurology and Neurosurgery, University of Florida, Gainesville; 12 Department of Neurology and Experimental Neurology, Charité–Universitätsmedizin Berlin, corporate member of Freie Universität Berlin and Humboldt–Universität zu Berlin, Berlin, Germany; 13 Department of Emergency Medicine, University of Michigan Medical School, Ann Arbor; 14 Department of Intensive Care, Emergency Medicine and Anaesthesiology–Fondazione Policlinico Universitario A. Gemelli, IRCCS–Università Cattolica del Sacro Cuore, Rome, Italy; 15 Institute of Anaesthesiology and Intensive Care Medicine, Università Cattolica del Sacro Cuore, Rome, Italy; 16 Department of Neurology, Boston University School of Medicine, Boston, Massachusetts; 17 MaineHealth Institute for Research, Scarborough; 18 Department of Critical Care Services, Maine Medical Center, Portland; 19 Tufts University School of Medicine, Boston, Massachusetts; 20 Department of Intensive Care, Hôpital Universitaire de Bruxelles, Université Libre de Bruxelles, Brussels, Belgium; 21 Department of Emergency, Critical Care, and Disaster Medicine, Okayama University Faculty of Medicine, Dentistry, and Pharmaceutical Sciences, Okayama, Japan; 22 Southmead Hospital, North Bristol NHS Trust, Bristol, United Kingdom; 23 Nantes Université, Nantes University Hospital, Médecine Intensive Reanimation, Motion-Interactions-Performance Laboratory, UR 4334, Nantes, France; 24 Warwick Clinical Trials Unit, University of Warwick, Warwick, United Kingdom; 25 Department of Anaesthesia and Intensive Care Medicine, Royal United Hospitals Bath NHS Foundation Trust, Combe Park, Bath, United Kingdom; 26 Department of Neurology, Stanford University, Palo Alto, California; 27 Division of Pulmonary, Critical Care and Sleep, Beth Israel Deaconess Medical Center, Boston, Massachusetts; 28 Division of Neurology and Rehabilitation, Department of Clinical Sciences, Lund University, Skåne University Hospital, Lund, Sweden; 29 Department of Anaesthesia and Intensive Care, Helsinki University Hospital and University of Helsinki, Helsinki, Finland; 30 Section of Emergency Medicine, Department of Medicine, University of Chicago, Chicago, Illinois; 31 CyrenCare, Inc, Vancouver, Washington; 32 Vancouver General Hospital, Department of Medicine, Faculty of Medicine, University of British Columbia, Vancouver, Canada; 33 Department of Emergency Medicine and Division of Pulmonary, Critical Care, and Sleep Medicine, University of Washington, Seattle; 34 Instituto Estadual do Cérebro Paulo Niemeyer, Rio de Janeiro, Brazil; 35 Johns Hopkins University School of Medicine, Baltimore, Maryland; 36 Department of Neurology, Columbia University Irving Medical Center, New York, New York; 37 Institute of Clinical Medicine, University of Oslo, Oslo, Norway; 38 Department of Anesthesia and Intensive Care Medicine, Oslo University Hospital, Oslo, Norway

## Abstract

**Importance:**

Understanding the relationship between patients’ clinical characteristics and outcomes is fundamental to medicine. When critically ill patients die after withdrawal of life-sustaining therapy (WLST), the inability to observe the potential for recovery with continued aggressive care could bias future clinical decisions and research.

**Objective:**

To quantify the frequency with which experts consider patients who died after WLST following resuscitated cardiac arrest to have had recovery potential if life-sustaining therapy had been continued.

**Design, Setting, and Participants:**

This prospective cohort study included comatose adult patients (aged ≥18 years) treated following resuscitation from cardiac arrest at a single academic medical center between January 1, 2010, and July 31, 2022. Patients with advanced directives limiting critical care or who experienced cardiac arrest of traumatic or neurologic etiology were excluded. An international cohort of experts in post-arrest care based on clinical experience and academic productivity was identified. Experts reviewed the cases between August 24, 2022, and February 11, 2024.

**Exposure:**

Patients who died after WLST.

**Main Outcome and Measures:**

Three or more experts independently estimated recovery potential for each patient had life-sustaining treatment been continued, using a 7-point numerical ordinal scale. In the primary analysis, which involved the patient cases with death after WLST, a 1% or greater estimated recovery potential was considered to be clinically meaningful. In secondary analyses, thresholds of 5% and 10% estimated recovery probability were explored.

**Results:**

A total of 2391 patients (median [IQR] age, 59 [48-69] years; 1455 men [60.9%]) were included, of whom 714 (29.9%) survived to discharge. Cases of uncertain outcome (1431 patients [59.8%]) in which WLST preceded death were reviewed by 38 experts who rendered 4381 estimates of recovery potential. In 518 cases (36.2%; 95% CI, 33.7%-38.7%), all experts believed that recovery potential was less than 1% if life-sustaining therapies had been continued. In the remaining 913 cases (63.8%; 95% CI, 61.3%-66.3%), at least 1 expert believed that recovery potential was at least 1%. In 227 cases (15.9%; 95% CI, 14.0%-17.9%), all experts agreed that recovery potential was at least 1%, and in 686 cases (47.9%; 95% CI, 45.3%-50.6%), expert estimates differed at this threshold.

**Conclusions and Relevance:**

In this cohort study of comatose patients resuscitated from cardiac arrest, most who died after WLST were considered by experts to have had recovery potential. These findings suggest that novel solutions to avoiding deaths based on biased prognostication or incomplete information are needed.

## Key Points

**Question:**

How often do experts believe that there was recovery potential in patients who had limitations or withdrawal of life-sustaining therapy (WLST) after resuscitation from cardiac arrest?

**Findings:**

In this cohort study of 2391 comatose survivors of cardiac arrest, all cases of patients who died after WLST (1431 [59.8%]) were evaluated. Upon independent review by 38 international experts, 913 patients (63.8%) who died after WLST were believed to have had a chance of recovery had life-sustaining therapy been continued.

**Meaning:**

These findings suggest that pessimistic clinical impressions and treatment decisions may perpetuate therapeutic nihilism and biased research.

## Teaser

This cohort study examines the frequency with which experts consider comatose patients who died after withdrawal of life-sustaining therapy following cardiac arrest to have had recovery potential had life-sustaining therapy continued.

## Introduction

Understanding the relationship between clinical characteristics and/or treatments and subsequent patient outcomes is fundamental to medicine.^1^ To elucidate these relationships, both observational studies^2^ and trials^3^ have relied on the strong assumption that outcomes are observed in a reliable and unbiased fashion.^4^ For critically ill patients,^5^ especially those resuscitated from cardiac arrest,^6,7^ death most often occurs after decisions to limit, forgo, or withdraw life-sustaining therapies. These decisions are based on beliefs that outcomes would be poor even if treatments continued. However, patients’ potential to recover, given their clinical characteristics and treatments, cannot be observed after withdrawal of life-sustaining therapy (WLST).^8,9^

**Figure 1.**
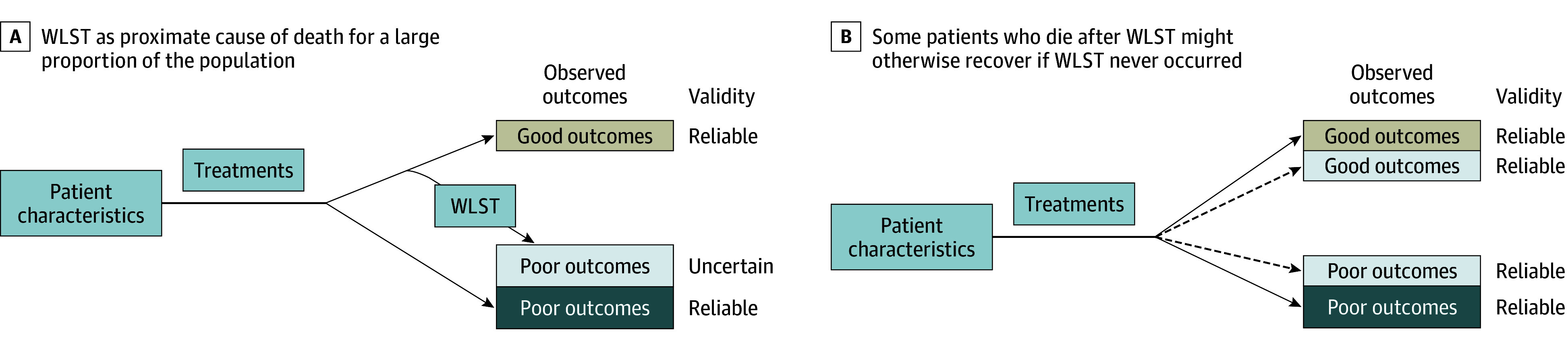
Association of Clinical Characteristics and Treatments With Patient Outcomes for Making Rational Medical Decisions and Advancing Science Dashed lines indicate counterfactual theoretical outcomes. Colors map between panels to show the same patients. WLST indicates withdrawal of life-sustaining therapy.

Critical care research that does not account for WLST is limited in that it can only identify the association among clinical characteristics, WLST, and observed outcomes (Figure 1), which may be biased if WLST is not limited to patients who would die regardless. Using these estimates may create, sustain, and/or amplify self-fulfilling prophecies, in which clinical findings prompt WLST, and deaths observed after WLST reinforce prior beliefs that prognoses were poor.^10^ In this setting, the true natural history of disease and recovery trajectories remain largely unknown.

Various approaches have been used to address this problem. Most research has assumed that all patients with WLST would have had poor outcomes, but this requires clinical decisions and prognostic testing to be perfectly accurate. It is known that prognostication is imperfect. Assuming that all patients who have WLST very early would have recovered with longer support is also too extreme^11^ because many patients who die after WLST may have had poor outcomes even with continued support. Some studies have tried to minimize WLST by standardizing prognostication and delaying WLST. However, the reported incidence of early WLST in deviation from recommendations is high.^12^ Alternatively, studies might exclude patients with WLST from analysis, which may lead to the assumption that the remaining cohort is representative. Because WLST is informed by observed clinical characteristics, excluding patients who die after WLST may sacrifice the representative nature of the cohort. This approach also may create results that cannot be applied prospectively, when patients’ future WLST status is unknown. Some research has been conducted in regions where WLST is either legally prohibited or culturally eschewed,^13,14,15^ but perceptions of poor prognosis may still reduce intensity of treatments believed to be ineffective.

We speculated that many patients resuscitated from cardiac arrest who died after WLST had recognizable, irrecoverable illness and, thus, could safely be considered to have had poor outcomes, while others who died after WLST had potential for recovery, and so could create bias if treated as if they had poor outcomes. We tested a novel approach to address this challenge using groups of experts to adjudicate probable outcomes with continued aggressive support for patients who died after WLST. We aimed to estimate the frequency with which decedents after WLST would be considered by experts to have had a chance of recovery if life-sustaining therapy had been continued.

## Methods

### Patients and Setting

This cohort study was approved by the University of Pittsburgh Human Research Protection Office with a waiver of informed consent for no greater than minimal risk research activities. We followed the Strengthening the Reporting of Observational Studies in Epidemiology (STROBE) reporting guideline.

We performed this study using data from a prospective, single-center registry of adults (aged ≥18 years) who were unresponsive to verbal commands after resuscitation from cardiac arrest and hospitalized between January 1, 2010, and July 31, 2022. We excluded patients who died after WLST based solely on preexisting advanced directives since prognostication does not guide this decision. We further excluded patients who experienced a cardiac arrest from trauma or neurologic etiologies because prognostication in these patients differs greatly from the general post-arrest cohort. We also excluded patients who did not survive to hospital admission, as reliable clinical information was inconsistently available.

We divided patients into 3 mutually exclusive groups based on their clinical outcome and circumstances of death, which were recorded prospectively.^16^ We considered patients to have good outcomes if they regained consciousness and survived to hospital discharge, corresponding to a Cerebral Performance Category (CPC) of 1 to 3. Many cardiac arrest studies have considered CPC 3 (conscious but dependent on others) assessed at 90 or 180 days to be a poor outcome. We considered CPC 3 at discharge to be a good outcome because function improves over the months after discharge from acute care in most patients after cardiac arrest.^17^ We considered patients to have poor outcomes if they were discharged in a persistently unresponsive state (CPC 4) or progressed to death by neurologic criteria. The remaining patients had uncertain outcomes and died either after WLST or because of worsening multisystem organ failure or rearrest. We included this latter group because unmeasured treatment decisions may have limited the intensity of aggressive care (eg, a decision not to offer mechanical circulatory support or kidney replacement therapy). We reviewed uncertain outcome cases in depth to obtain experts’ estimates of recovery potential.

### Expert Case Review

We assembled a group of international experts in post-arrest care and prognostication. We defined expertise as meeting at least 2 of 3 criteria: (1) cared for >150 patients after a cardiac arrest, (2) published more than 3 peer-reviewed publications related to post-arrest care and prognostication within 3 years (2019-2022), and (3) played a leadership role in a large trial of post-arrest care. For each uncertain outcome case, we prepared a deidentified structured presentation with the full available information. We scheduled groups of 3 to 4 experts for 1-hour video conference case reviews, during which we presented structured vignettes and the original neuroimaging and electroencephalograms. Experts could ask the presenter, who had access to the electronic health record, for additional or clarifying information.

After all questions were answered, we asked experts to estimate independently the outcome for the patient in response to the following prompt: “If life-sustaining therapies had been continued, what would be this patient’s probability of regaining consciousness and surviving hospital discharge?” We pilot tested with 7 experts various methods to elicit responses, including a Likert scale, slide bar, and numeric percentage response. After obtaining and analyzing their qualitative feedback on each strategy, we judged content, construct, face validity, and intrarater consistency to be the best using a numerical ordinal scale (Table 1). This ordinal response scale provided verbal qualifiers natural to clinical users and grounded these in numeric equivalents. Experts also recorded their confidence in their response on a 6-point Likert scale ranging from completely uncertain to completely certain.

**Table 1.** Estimated Recovery Potential of Patients Who Died After WLST for Perceived Poor Neurologic Prognosis and/or Succumbing to Multisystem Organ Failure or Rearrest

Response category	Estimate of patient’s probability of being awake and alive at hospital discharge (CPC 1-3), %^a^
1	0, No chance of awakening and survival to discharge
2	>0 to 1, Trivial chance of awakening and survival
3	>1 to 5, Very small chance of awakening and survival
4	>5 to 10, Small chance of awakening and survival
5	>10 to 25, Moderate chance of awakening and survival
6	>25 to 50, Good chance of awakening and survival
7	>50, More likely than not to awaken and survive

Abbreviations: CPC, Cerebral Performance Category; WLST, withdrawal of life-sustaining therapy.

^a^ After a detailed case review, the experts were prompted to estimate recovery potential on a numerical ordinal scale designed to reflect clinically relevant probability categories.

We reviewed all uncertain outcome cases, and experts were aware of patients’ observed outcomes. To assess test-retest reliability, we presented 34 random cases a second time to the same experts who completed a first case review. To minimize recall, this second round occurred at least 1 year after the first presentation, and we did not disclose the repeat nature of the assessment. To assess calibration, we integrated 30 random good outcome cases. For these, we presented information until hospital discharge but concealed actual outcomes without disclosing that these cases differed from the typical case review. Experts reviewed the cases between August 24, 2022, and February 11, 2024.

### Statistical Analysis

We summarized clinical characteristics overall and by group. We did not collect data on patient race and ethnicity because these could not be ascertained reliably from the electronic health record. The remaining analyses focused on the uncertain outcome group. We summarized expert responses using descriptive statistics and compared their distribution across circumstances of death using a Wilcoxon rank sum test. We compared the distribution of responses stratified by expert practice location (US vs international, treating hospital vs elsewhere) to test whether familiarity with local practice patterns affected responses. We also compared the distribution of responses between deaths that occurred less than 72 hours after cardiac arrest vs 72 hours or longer after the arrest.

In our primary analysis, we determined the proportion of uncertain outcome cases in which at least 1 expert believed there was at least a 1% probability of a good outcome if life-sustaining therapy had been continued and calculated the binomial confidence interval around this point estimate. We chose to dichotomize responses at this threshold on the basis of our group’s prior research in which international medical stakeholders identified this cutoff as clinically relevant.^18^

We then divided cases into 3 categories: (1) all experts agreed there was a less than 1% chance of a good outcome if life-sustaining therapy had been continued; (2) all experts agreed that there was at least a 1% chance of a good outcome; and (3) experts’ responses differed at the 1% threshold. In a secondary analysis, we defined differing responses as cases where expert responses varied by at least 2 or at least 3 levels in the ordinal response scale (Table 1). In an additional secondary analysis, we dichotomized responses at thresholds of 5% and 10% estimated recovery probability.

To explore variability among experts, we determined the median expert response for each case, then calculated the proportion of cases for which each expert agreed with the median response, estimated a higher chance of recovery, or estimated a lower chance of recovery than the median. We collected expert responses using Research Electronic Data Capture software (REDCap) and performed analyses using R, version 4.4.1 (R Foundation). The threshold for significance was *P* < .05.

## Results

**Figure 2.**
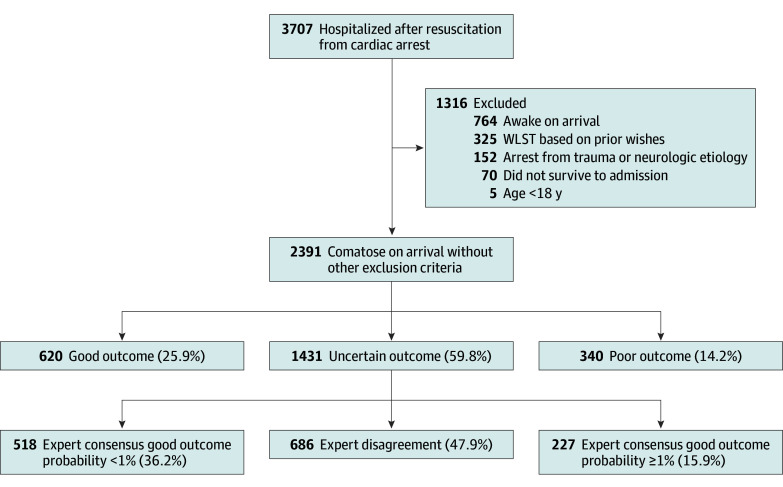
Flow Diagram for the Study Cohort WLST indicates withdrawal of life-sustaining therapy.

During the study period, we treated 3707 patients resuscitated after cardiac arrest, of whom 1316 were excluded (Figure 2). Of 2391 included patients (median [IQR] age, 59 [48-69] years; 1455 men [60.9%] and 936 women [39.1%]), 714 (29.9%) survived to discharge. Most patients (1431 [59.8%]) were in the uncertain outcome group, 620 (25.9%) were in the good outcome group, and 340 (14.2%) were in the poor outcome group (94 [27.6%] were discharged with CPC 4 and 246 [72.4%] died by neurologic criteria). As expected, characteristics differed by group (Table 2). Time from cardiac arrest to WLST for perceived poor neurologic prognosis was 76 hours (IQR, 37-127 hours), time from cardiac arrest to death from multisystem organ failure was 15 hours (IQR, 6-53 hours), and time from arrest to awakening in the good outcome group was 47 hours (IQR, 34-91 hours).

**Table 2.** Cohort Characteristics Overall and Stratified by Outcome Group

Characteristic	Patients, No. (%)			
	Overall cohort (N = 2391)	Poor outcome observed (n = 340)	Good outcome observed (n = 620)	Uncertain outcome (n = 1431)
Age, median (IQR), y	59 (48-69)	51 (36-61)	58 (48-67)	62 (51-71)
Sex				
Female	936 (39.1)	159 (46.8)	211 (34.0)	566 (39.6)
Male	1455 (60.9)	181 (53.2)	409 (66.0)	865 (60.4)
Pittsburgh Cardiac Arrest Category				
II	537 (22.5)	46 (13.5)	329 (53.1)	162 (11.3)
III	263 (11.0)	19 (5.6)	107 (17.3)	137 (9.6)
IV	1251 (52.3)	237 (69.7)	63 (10.2)	951 (66.5)
Unknown	340 (14.2)	38 (11.2)	121 (19.5)	181 (12.6)
Out-of-hospital arrest	1974 (82.6)	316 (92.9)	484 (78.1)	1174 (82.0)
Initial arrest rhythm				
VT/VF	669 (28.0)	54 (15.9)	311 (50.2)	304 (21.2)
PEA	845 (35.3)	109 (32.1)	196 (31.6)	540 (37.7)
Asystole	734 (30.7)	153 (45.0)	80 (12.9)	501 (35.0)
Unknown	143 (6.0)	24 (7.1)	33 (5.3)	86 (6.0)
Witnessed collapse^a^	1167 (59.1)	167 (58.2)	292 (60.3)	708 (60.3)
Layperson CPR^a^	1326 (67.2)	244 (77.2)	300 (62.0)	782 (66.6)
Arrest duration, median (IQR), min	20 (11-31)	26 (17-40)	11 (6-19)	22 (14-33)
Target temperature				
33 °C	1084 (45.3)	153 (45.0)	231 (37.3)	700 (48.9)
36 °C	827 (34.6)	99 (29.1)	278 (44.8)	450 (31.4)
Other target	189 (7.9)	37 (10.9)	45 (7.3)	107 (7.5)
No TTM	291 (12.2)	51 (15.0)	66 (10.6)	174 (12.2)
Coronary angiography	486 (20.3)	37 (10.9)	263 (42.4)	186 (13.0)
Electroencephalography	1837 (76.8)	253 (74.4)	497 (80.2)	1087 (76.0)
Brain computed tomography	1932 (80.8)	323 (95.0)	479 (77.3)	1130 (79.0)
GWR, median (IQR)	1.31 (1.21-1.37)	1.17 (1.01-1.31)	1.36 (1.31-1.41)	1.30 (1.20-1.36)
Arrest etiology				
Cardiac	702 (29.4)	54 (15.9)	302 (48.7)	346 (24.2)
Respiratory	528 (22.1)	91 (26.8)	103 (16.6)	334 (23.3)
Other	622 (26.0)	137 (40.3)	133 (21.5)	352 (24.6)
Uncertain	539 (22.5)	58 (17.1)	82 (13.2)	399 (27.9)
Outcome				
Survived	714 (29.9)	94 (27.6)	620 (100)	0
Brain death	246 (10.3)	246 (72.4)	0	0
MSOF	564 (23.6)	0	0	564 (39.4)
WLST-N	867 (36.3)	0	0	867 (60.6)

Abbreviations: CPR, cardiopulmonary resuscitation; GWR, gray matter to white matter radiodensity ratio; MSOF, multisystem organ failure; PEA, pulseless electrical activity; TTM, targeted temperature management; VT/VF, ventricular tachycardia/ventricular fibrillation; WLST-N, withdrawal of life-sustaining therapy for perceived poor neurologic prognosis.

^a^ Percentages expressed for the proportion of patients who had a cardiac arrest outside the hospital.

**Figure 3.**
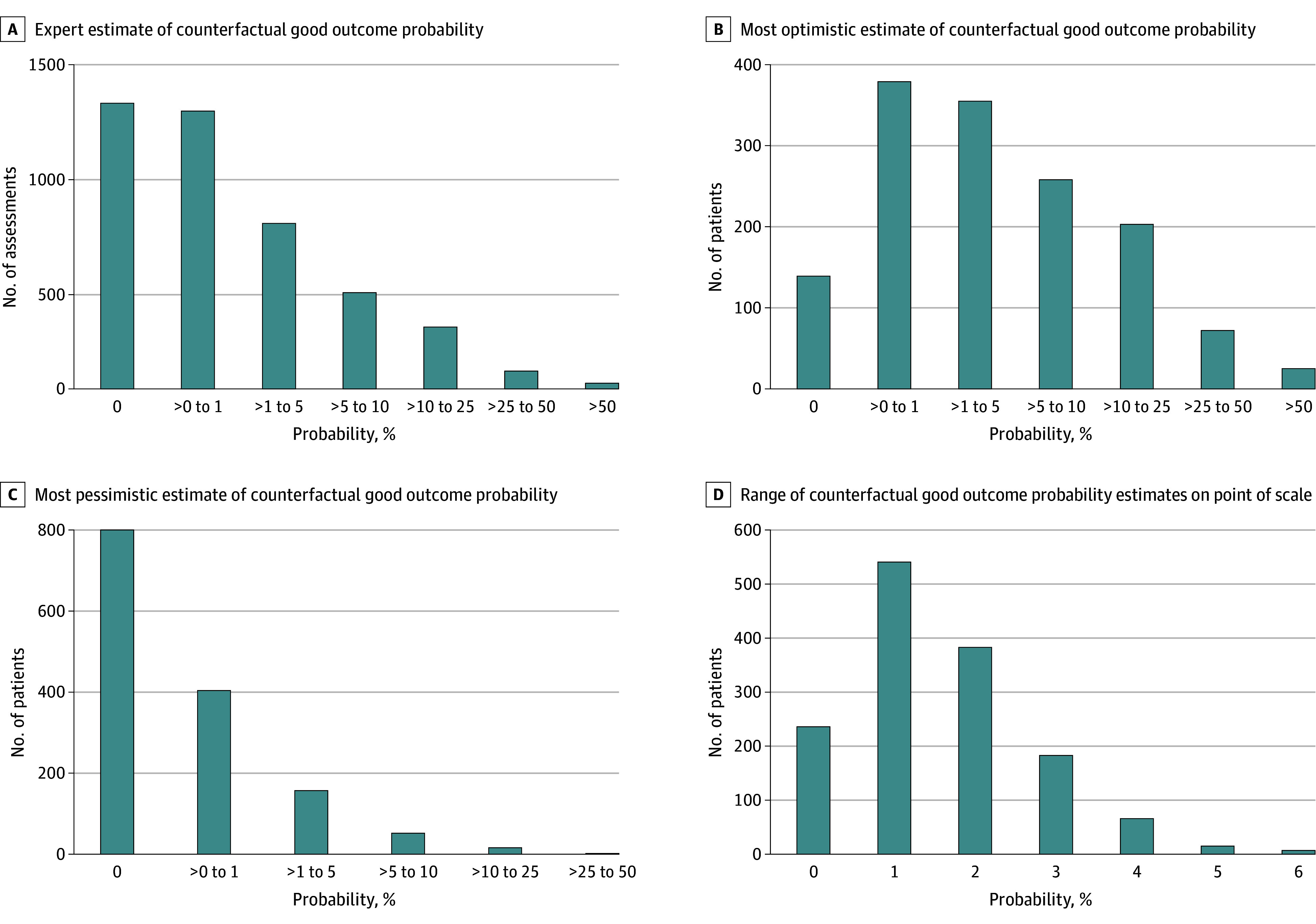
Distribution of Expert Estimates

We collected 4381 expert estimates of recovery potential from 38 experts during 1032 expert-hours of case review. Experts reviewed a median of 71 cases (IQR, 47-116 cases). The median expert outcome estimate was 0% to less than 1% (IQR, 0% to >1%-5%) (Figure 3A), and the median confidence was very certain (IQR, somewhat certain to completely certain) (eTable 1 and eFigure 1 in Supplement 1). Responses of experts practicing at the treating hospital were lower than those practicing elsewhere (eFigure 2A in Supplement 1). Outcome estimations were lower when death occurred less than 72 hours after cardiac arrest (eFigure 2B in Supplement 1).

### Expert Agreement

In 518 of the 1431 patient cases with uncertain outcomes because of WLST (36.2%; 95% CI, 33.7%-38.7%), all experts agreed that recovery potential was less than 1% if life-sustaining therapy had been continued, while in 913 (63.8%; 95% CI, 61.3%-66.3%), at least 1 expert believed that recovery potential was at least 1% (Figure 3B). In 227 cases (15.9%; 95% CI, 14.0%-17.9%), all experts agreed that recovery potential was at least 1% if life-sustaining therapy had been continued (Figure 3C). In the remaining 686 (47.9%; 95% CI, 45.3%-50.6%), expert responses differed at the less than 1% vs 1% or greater threshold. Median expert confidence was lower in cases of disagreement (very confident; IQR, somewhat confident to very confident) compared with in cases of agreement (very confident; IQR, somewhat confident to completely confident; *P* < .001) (eFigure 1 in Supplement 1). The frequency with which expert responses differed did not vary by circumstances of death; expert confidence also did not vary by circumstances of death. In 654 cases (45.7%; 95% CI, 43.1%-48.3%), the range of divergence between expert responses was at least 2 levels on the ordinal response scale, and in 271 cases (18.9%; 95% CI, 16.9%-21.0%), the range of responses was at least 3 levels (Figure 3D).

In our secondary analysis examining other thresholds, all experts agreed that recovery potential was less than 5% in 988 of the 1431 cases (69.0%) they analyzed overall, while in 443 cases (31.0%), at least 1 expert estimated that recovery potential was at least 5% (agreement in 70 cases [15.8%] and differing responses in 373 cases [84.2%]). All experts agreed that recovery potential was less than 10% in 1228 cases (85.8%), while in 203 (14.2%), at least 1 expert estimated that recovery potential was at least 10% (agreement in 18 cases [8.9%] and differing responses in 185 cases [91.1%]).

### Between-Expert Variability

Because differing responses between experts within cases were common, individual expert responses often differed from the median response for each case (eFigure 4 in Supplement 1). One expert’s estimations of recovery potential were lower than the median in 9 of 12 of cases (75%) reviewed by that expert, deviating by a mean (SD) of −1.0 (0.8) level on the ordinal response scale. Two experts estimated a higher recovery potential than the median in 16 of 28 cases (57%) and 20 of 26 cases (77%) they reviewed, deviating by a mean (SD) of 1.1 (1.3) and 1.2 (1.3) levels on the ordinal response scale, respectively. Individual responses of the remaining 35 experts compared with the case medians were normally distributed. No results significantly differed when the 3 outlier experts’ assessments were excluded from the analysis.

### Intrarater Reliability and Calibration

In 34 test-retest assessments, intrarater reliability was strong (median within-expert difference, 0 levels; IQR, 0-1 levels) on the ordinal response scale. In 30 assessments of expert calibration to detect observed good outcomes, the median outcome estimate was the highest recovery potential of more likely than not to awaken and survive (>50%), and the most pessimistic estimate was good chance of awakening and survival (>25% to 50%) (eTable 2 in Supplement 1).

## Discussion

This cohort study’s major findings were that for most comatose patients resuscitated from cardiac arrest who died after WLST, at least 1 expert believed that there was a chance of recovery if life-sustaining treatment had been continued, and in only one-half of these cases, experts agreed about recovery potential. Although we focused on post-arrest care as a specific example of critical illness, the frequency with which at least 1 expert believed that patients who died after WLST had a chance of recovery (36.2% of the overall cohort) highlights an epistemic problem in critical care relevant to many patient populations. Biomedical discovery may be stymied when individual treatment decisions are simultaneously influenced by clinical characteristics and deterministic of subsequent outcomes.

This problem is relevant to both clinicians and researchers. Clinical decision-making, including post-arrest prognostication, is cognitively complex.^19^ Novice clinicians develop expertise by recalibrating their judgments through experience and feedback, eventually developing heuristics upon which they rely.^20,21,22^ Deaths after WLST for perceived poor prognosis reinforce prior clinicians’ beliefs that prognosis was poor; after all, these patients were observed to die, and hence, estimations of poor prognosis were empirically correct. This cycle may lead to poorly calibrated judgments and therapeutic nihilism and may perpetuate systemic and systematic biases.^10^ Although consensus statements and treatment guidelines seek to mitigate these risks and urge caution when estimating prognosis,^23^ research has suggested that simply warning clinicians about risks of bias is ineffective.^24^ Indeed, research of post-arrest care in the US has suggested that most patients die before an outcome can be estimated reliably and in the absence of guideline-concordant prognostic assessments.^25^

From a research perspective, knowledge is uncertain when generated in settings where WLST is common.^26^ Current approaches that seek to minimize resulting bias are inadequate. The most conservative approach is to perform sensitivity analyses in which patients who died after WLST are considered to have potentially favorable outcomes. In our cohort, this approach would have increased the apparent frequency of good outcomes from 26% to 86%. While such findings may offer bounds on the range of potential results,^27^ they are so broad as to obviate their usefulness, particularly in prognostication tasks where clinicians demand stringent performance metrics.^18^

We considered a probability of at least 1% of regaining consciousness and surviving to hospital discharge to be important based on previous research.^18^ When we considered higher thresholds, differing responses between experts decreased but remained common. From a clinical perspective, surrogates can make ethically appropriate, patient-centered decisions for WLST even when recovery is believed possible. These decisions may be motivated by concerns about achieving an acceptable quality of recovery, long-term prognosis, or tolerability of continued critical care in the face of an uncertain outcome. Our results do not speak to the appropriateness of individual WLST decisions but, rather, highlight the challenge of providing surrogates with reliable prognoses to inform these high-stakes decisions.

Our work offers a path forward, in which groups of experts can estimate potential outcomes. With care, this knowledge could be incorporated into prognostic models. The best method to achieve this subsequent step is an area of ongoing research. Analyzing pooled responses from experts treats both the experts and their responses as equivalent. However, experts may formulate their prognostic judgments based on fundamentally different views, making the average expert judgment a nonsensical quantity. Depending on the application, projections from the most optimistic and pessimistic experts might provide a credible range of estimates. However, even after more than 1000 review hours, most experts did not review most cases, and accounting for potentially undesirable between-expert variability (eFigures 3 and 4 in Supplement 1) is mandatory. The best strategy to elicit expert judgments is also a knowledge gap.^28^ Our use of a numerical ordinal scale may have introduced response range effects, whereby some respondents may have felt compelled to use the full range of response options. However, although both expert responses and agreement between experts were imperfect, they differed somewhat in their limitations, providing converging forms of evidence.

### Limitations

This study had several limitations. Although we believe our findings to be relevant broadly in critical care, we have described results from a single-center cohort of patients resuscitated after cardiac arrest. The rate and circumstances of death in our single-center study resemble that in prior multicenter cohorts examining outcomes of post-arrest care,^6,7^ suggesting that our results may generalize to many settings. A greater limitation is that no outcome data in the absence of WLST were available against which to validate our findings. Experts were aware of the circumstances of death in the uncertain outcome cases and themselves developed clinical expertise without feedback. These factors may have influenced their estimates. Responses of experts practicing at the treating center were slightly lower than the responses of experts practicing elsewhere (eFigure 2A in Supplement 1), though this may also reflect greater uncertainty from experts who were less familiar with the clinical environment in which patients were treated. While experts were able to reliably identify patients who had favorable outcomes, we cannot reliably assess their calibration in the bulk of cases. Additionally, we considered patients with CPC 4 at hospital discharge to have had poor outcomes, but some may have recovered consciousness after hospital discharge or had cognitive-motor dissociation and unrecognized consciousness on discharge.

## Conclusions

In this cohort study of comatose survivors of cardiac arrest, most patients who died after WLST were considered by experts to have had recovery potential. Our results suggest a potential for biased clinical decision-making and research when deaths after WLST are treated as poor outcomes that cannot be ignored. While in some cases experts agreed that recovery prospects were less than 1%, in many others, experts estimated substantial recovery potential. As a community of clinicians and scientists, we must develop better approaches to mitigate the risk of self-fulfilling prophecies and preventable deaths.

## Notes

Supplement 1. **eTable 1. **Experts Rated Their Self-Confidence in Each Outcome Estimate on a 6-Level Ordinal Scale **eFigure 1.** Median Expert Confidence was Lower in Cases Where There Was Expert Disagreement About Recovery Potential at the ≤1% vs >1% Outcome Threshold **eTable 2.** Distribution of Expert Responses for Good Outcome Cases **eFigure 2.** Expert Responses Stratified by Location and Time From Arrest to Death **eFigure 3.** The Proportion of Cases Assessed by Each Expert in Which That Expert Estimated Either a Higher or Lower Recovery Probability than the Median Expert for that Case **eFigure 4.** The Average Deviation of Each Expert’s Estimate Compared With the Median of Each Case, in Levels of the Ordinal Response Scale

Supplement 2.
Nonauthor Collaborators

Supplement 3.
Data Sharing Statement
