# Supplementary material for: Recovery Potential in Patients After Cardiac Arrest Who Die After Limitations or Withdrawal of Life Support
Source: JAMA Netw Open. 2025 Mar 25;8(3):e251714. doi: 10.1001/jamanetworkopen.2025.1714 (PMC11937936; doi:10.1001/jamanetworkopen.2025.1714)
Supplement: Supplement 1. — eTable 1. Experts Rated Their Self-Confidence in Each Outcome Estimate on a 6-Level Ordinal Scale eFigure 1. Median Expert Confidence was Lower in Cases Where There Was Expert Disagreement About Recovery Potential at the ≤1% vs >1% Outcome Threshold eTable 2. Distribution of Expert Responses for Good Outcome Cases eFigure 2. Expert Responses Stratified by Location and Time From Arrest to Death eFigure 3. The Proportion of Cases Assessed by Each Expert in Which That Expert Estimated Either a Higher or Lower Recovery Probability than the Median Expert for that Case eFigure 4. The Average Deviation of Each Expert’s Estimate Compared With the Median of Each Case, in Levels of the Ordinal Response Scale [file jamanetwopen-e251714-s001.pdf]

Supplemental Online Content

Elmer J, Coppler PJ, Ratay C, et al; Optimizing Recovery Prediction After Cardiac Arrest (ORCA) Study Group. Recovrey potential in patients after cardiac arrest who die after limitations or withdrawal of life support. *JAMA Netw Open*. 2025;8(3):e251714. doi:10.1001/jamanetworkopen.2025.1714

eTable 1. Experts Rated Their Self-Confidence in Each Outcome Estimate on a 6-Level Ordinal Scale
eFigure 1. Median Expert Confidence was Lower in Cases Where There Was Expert Disagreement About Recovery Potential at the ≤1% vs >1% Outcome Threshold
eTable 2. Distribution of Expert Responses for Good Outcome Cases
eFigure 2. Expert Responses Stratified by Location and Time From Arrest to Death
eFigure 3. The Proportion of Cases Assessed by Each Expert in Which That Expert Estimated Either a Higher or Lower Recovery Probability than the Median Expert for that Case
eFigure 4. The Average Deviation of Each Expert's Estimate Compared With the Median of Each Case, in Levels of the Ordinal Response Scale

This supplemental material has been provided by the authors to give readers additional information about their work.

© 2025 Elmer J et al. *JAMA Network Open*.

eTable 1. Experts Rated Their Self-Confidence in Each Outcome Estimate on a 6-Level Ordinal Scale

**Response level**	**Self-reported confidence**
-3	Completely uncertain
-2	Very uncertain
-1	Somewhat uncertain
1	Somewhat certain
2	Very certain
3	Completely certain

eFigure 1. Median Expert Confidence was Lower in Cases Where There Was Expert Disagreement About Recovery Potential at the ≤1% vs >1% Outcome Threshold

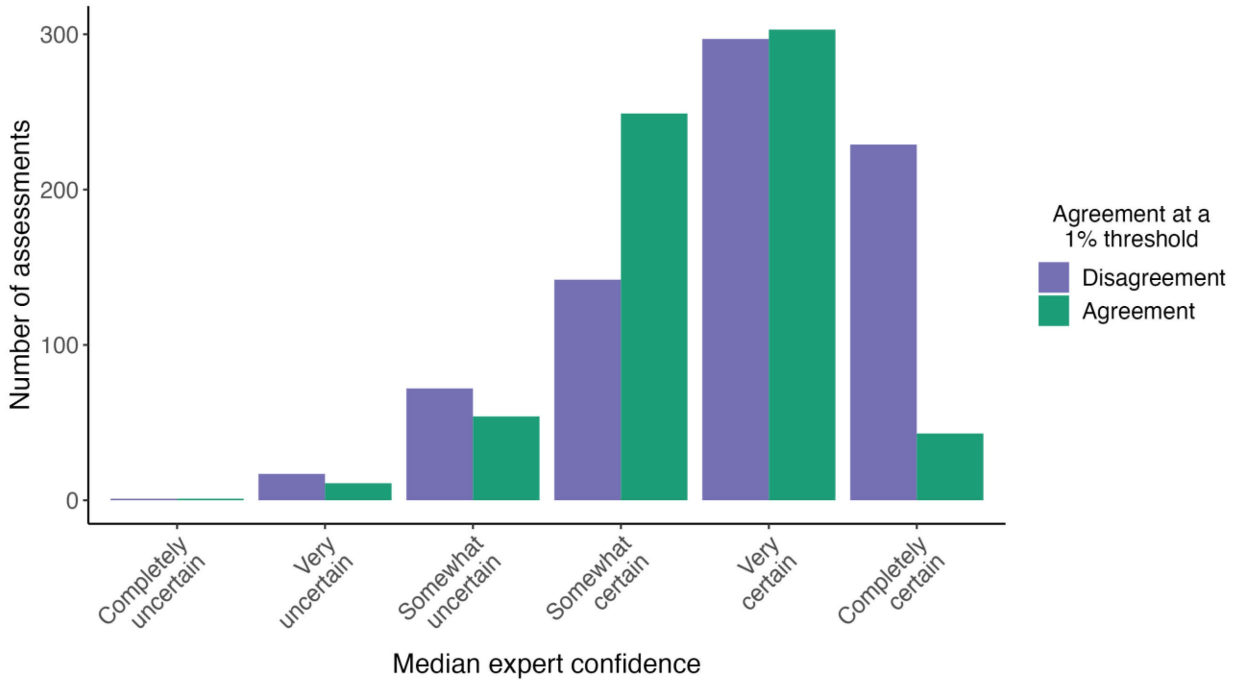

© 2025 Elmer J et al. *JAMA Network Open.*

eTable 2. Distribution of Expert Responses for Good Outcome Cases. Experts were blinded to the true observed outcome in their assessment.

Response category	If life-sustaining therapies had been continued, what do you estimate this patient's probability of being awake and alive at hospital discharge (i.e. discharge CPC 1-3) would have been?	Frequency (n = 90)
1	0% - No chance of awakening and survival to discharge	0
2	>0 to 1% - Trivial chance of awakening and survival	0
3	>1 to 5% - Very small chance of awakening and survival	0
4	>5 to 10% - Small chance of awakening and survival	0
5	>10 to 25% - Moderate chance of awakening and survival	0
6	>25 to 50% - Good chance of awakening and survival	24 (26.7%)
7	>50% - More likely than not to awaken and survive	66 (73.3%)

© 2025 Elmer J et al. *JAMA Network Open*.

eFigure 2. Expert Responses Stratified by Location and Time From Arrest to Death
A) Expert responses stratified by practice location (practiced at the hospital where included patients were treated vs practiced elsewhere). B) Expert responses stratified by time from arrest to death.

A.

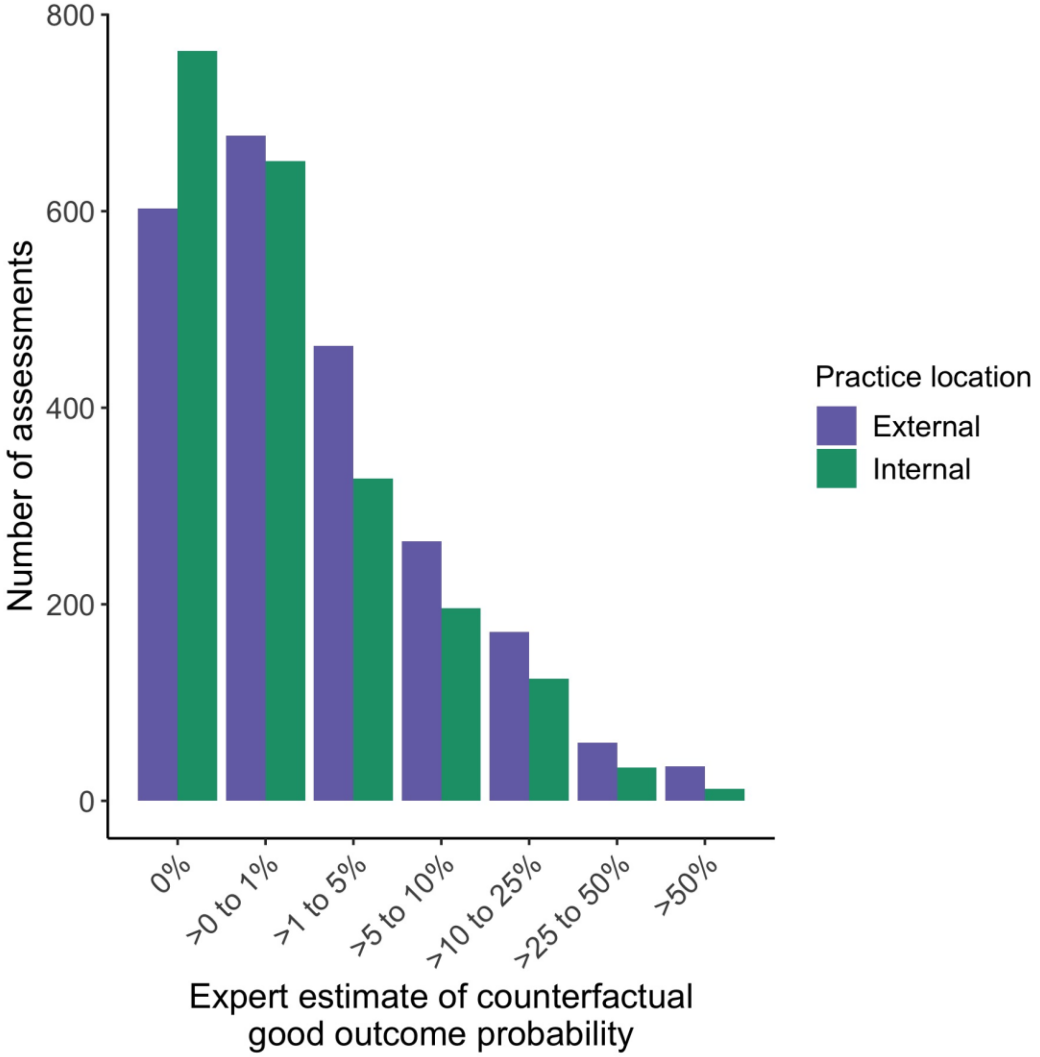

© 2025 Elmer J et al. *JAMA Network Open*.

B.

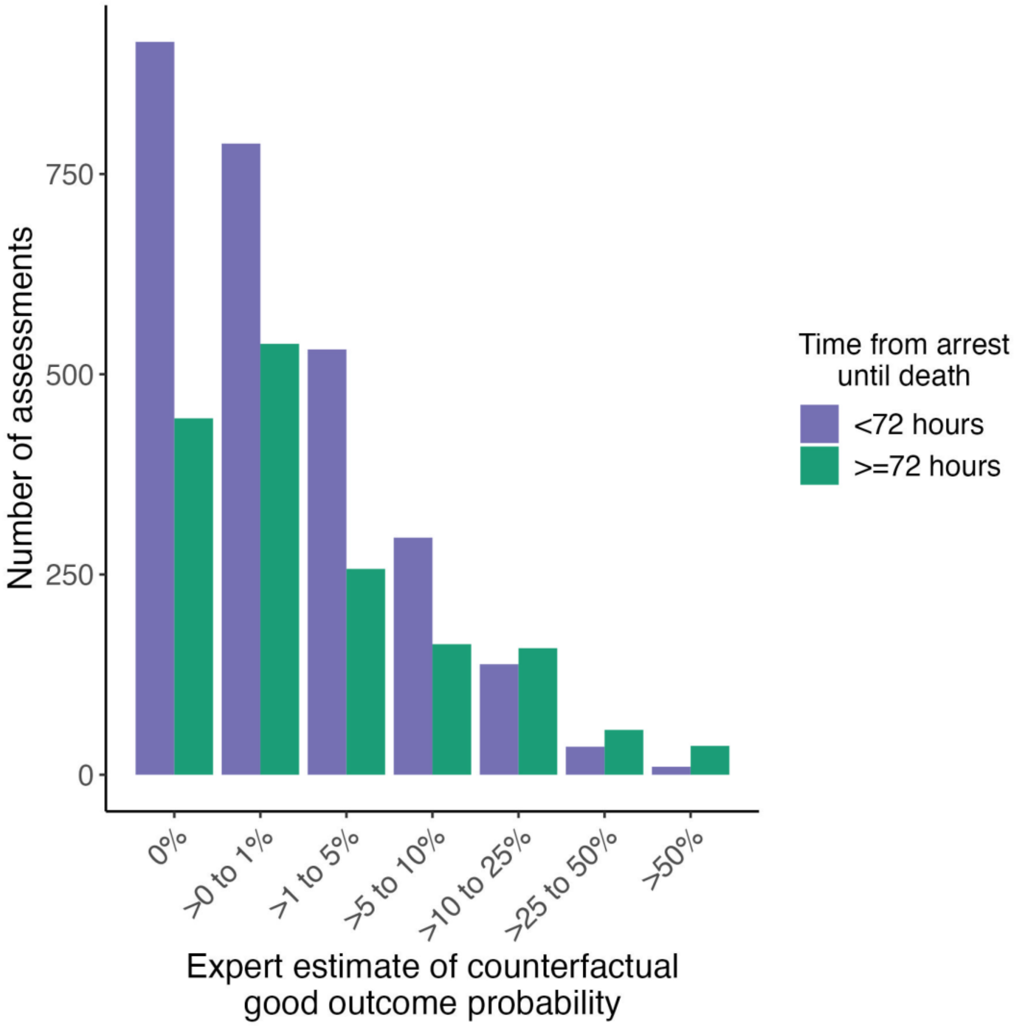

© 2025 Elmer J et al. *JAMA Network Open*.

eFigure 3. The Proportion of Cases Assessed by Each Expert in Which That Expert Estimated Either a Higher or Lower Recovery Probability than the Median Expert for that Case

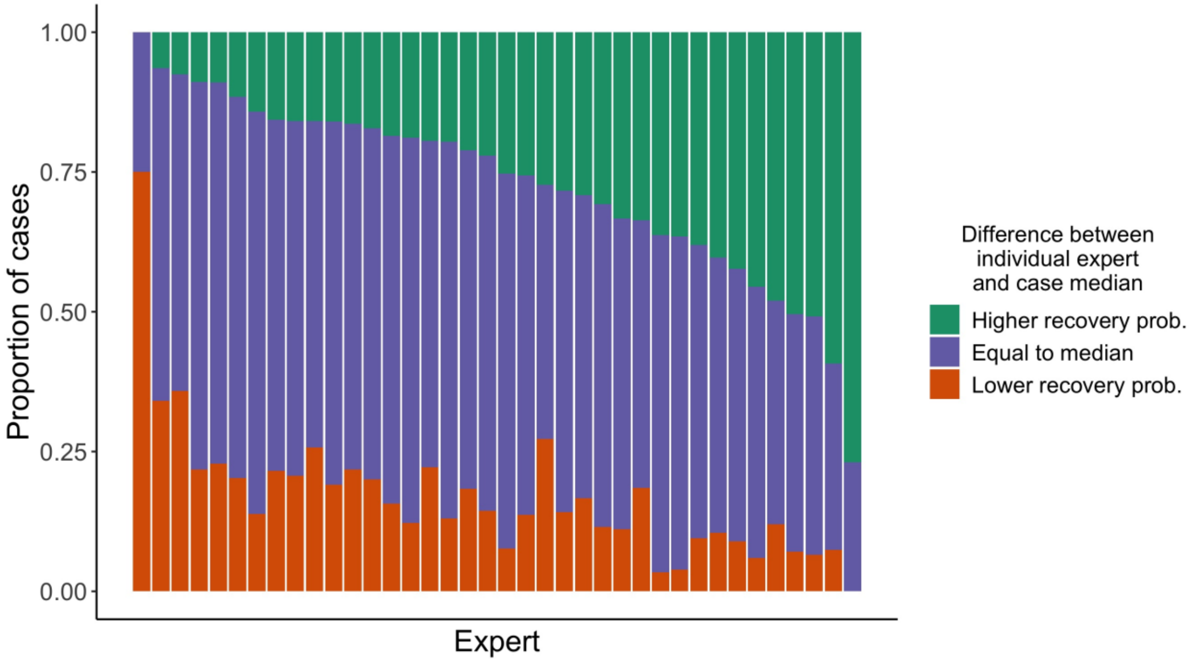

© 2025 Elmer J et al. *JAMA Network Open*.

eFigure4. The Average Deviation of Each Expert's Estimate Compared With the Median of Each Case, in Levels of the Ordinal Response Scale

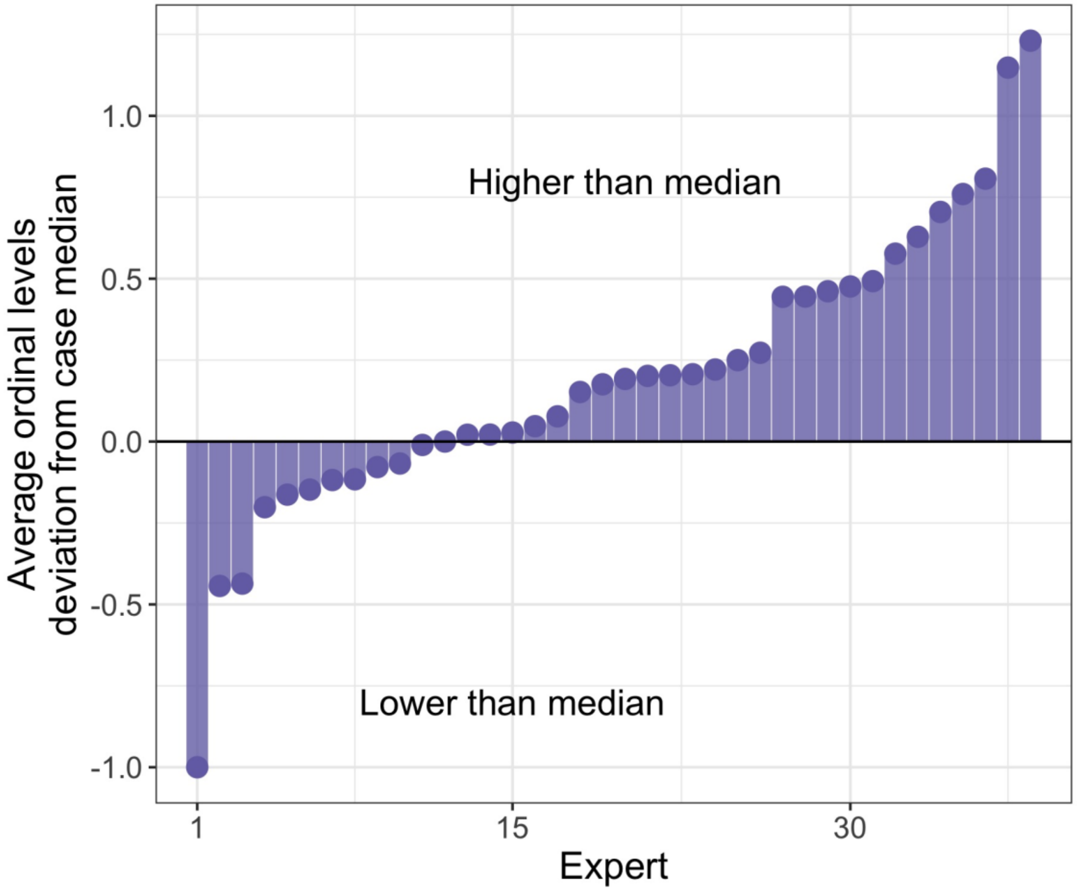

© 2025 Elmer J et al. *JAMA Network Open*.
