# Supplementary material for: Recovery Potential in Patients After Cardiac Arrest Who Die After Limitations or Withdrawal of Life Support
Source: JAMA Netw Open. 2025 Mar 25;8(3):e251714. doi: 10.1001/jamanetworkopen.2025.1714 (PMC11937936; doi:10.1001/jamanetworkopen.2025.1714)
Supplement: Supplement 2. — Nonauthor Collaborators [file jamanetwopen-e251714-s002.pdf]

Supplemental Online Content: Nonauthor Collaborators
*First name, last name, and suffix (if applicable) are required and will appear in PubMed.

*Group Name(s): the Optimizing Recovery prediction after Cardiac Arrest (ORCA) Study Group							
***First Name and Middle Initial(s)**	***Last Name**	***Suffix (eg, Jr, III)**	Academic Degrees	Institution	Location (city, state/province, country)	Role or Contribution, eg, chair, principal investigator	Group (if more than 1 Group listed in the byline) and/or Subgroup (eg, Steering Committee)
Alain	Cariou		MD, PhD	Médecine Intensive et Réanimation - Hôpital Cochin, APHP Centre - Université Paris Cité	Paris, France	Investigator	
Alejandro A.	Rabinstein		MD, PhD	Mayo Clinic	Rochester MN, USA	Investigator	
Alexis	Steinberg		MD, MS	University of Pittsburgh	Pittsburgh PA, USA	Investigator	
Andrea O.	Rossetti		MD	University Hospital (CHUV) and University of Lausanne	Lausanne, Switzerland	Investigator	
Ankur A.	Doshi		MD	University of Pittsburgh	Pittsburgh PA, USA	Investigator	
Bradley J.	Molyneaux		MD, PhD	Brigham and Women’s Hospital	Boston MA, USA	Investigator	
Cameron	Dezfulian		MD	Baylor College of Medicine	Houston TX, USA	Investigator	
Carolina B	Maciel		MD, MSCR	University of Florida	Gainesville FL, USA	Investigator	
Cecelia	Ratay		DNP, CRNP	University of Pittsburgh	Pittsburgh PA, USA	Investigator	
Christoph	Leithner		MD	Charité–Universitätsmedizin Berlin, corporate member of Freie Universität Berlin and Humboldt- Universität zu Berlin	Berlin, Germany	Investigator	
Cindy	Hsu		MD, PhD, N	University of Michigan Medical School	Ann Arbor, MI USA	Investigator	
Claudio	Sandroni		MD	Università Cattolica del Sacro Cuore and Fondazione Policlinico Universitario Agostino Gemelli- IRCCS	Rome, Italy	Investigator	
Clifton W.	Callaway		MD, PhD	University of Pittsburgh	Pittsburgh PA, USA	Investigator	
David M.	Greer		MD	Boston University Chobanian & Avedisian School of Medicine	Boston MA, USA	Investigator	
David B.	Seder		MD	Maine Medical Center and Tufts Univeristy School of Medicine	Portland ME, USA	Investigator	
Francis X.	Guyette		MD, MS, M	University of Pittsburgh	Pittsburgh PA, USA	Investigator	
Fabio Silvio	Taccone		MD, PhD	Hopital Erasme and Université Libre de Bruxelles	Brussels, Belgium	Investigator	
Hiromichi	Naito		MD, PhD	Okayama University Faculty of Medicine, Dentistry, and Pharmaceutical Sciences	Okayama, Japan	Investigator	
Jasmeet	Soar		MB BChir,	Southmead Hospital, North Bristol NHS Trust	Bristol, United Kingdom	Investigator	
Jean-Baptiste	Lascarrou		MD, PhD	Centre Hospitalier Universitaire de Nantes	Nantes, France	Investigator	
Jerry P.	Nolan		FRCA, FRCI	Royal United Hospital	Bath, United Kingdom	Investigator	
Jonathan	Elmer		MD, MS	University of Pittsburgh	Pittsburgh PA, USA	Principal Investigator	
Karen G	Hirsch		MD	Stanford University	Palo Alto CA, USA	Investigator	
Katherine	Berg		MD	Harvard Medical School	Boston MA, USA	Investigator	
Marion	Moseby-Knappe		MD, PhD	Skane University Hospital and Lund University	Lund, Sweden	Investigator	
Markus B.	Skrifvars		MD, PhD	University of Helsinki	Helsinki, Finland	Investigator	
Michael	Donnino		MD	Harvard Medical School	Boston MA, USA	Investigator	
Michael	Kurz		MD	University of Chicago	Chicago IL, USA	Investigator	
Min Jung Kathy	Chae		MD, MS	CyrenCare	Vancouver WA, USA	Investigator	
Mypinder	Sekhon		MD, PhD	Vancouver General Hospital and University of British Columbia	Vancouver, Canada	Investigator	
Nicholas J.	Johnson		MD	University of Washington	Seattle WA, USA	Investigator	
Patrick J.	Coppler		PA-C	University of Pittsburgh	Pittsburgh PA, USA	Investigator	
Pedro	Kurtz		MD, PhD	Instituto Estadual do Cérebro Paulo Niemeyer	Rio de Janeiro, Brazil	Investigator	
Romergryko G.	Geocadin		MD	Johns Hopkins University School of Medicine	Baltimore MD, USA	Investigator	
Sachin	Agarwal		MD, MPH	Columbia University Irving Medical Center	New York NY, USA	Investigator	
Teresa L.	May		DO	Maine Medical Center and Tufts Univeristy School of Medicine	Portland ME, USA	Investigator	
Theresa Mariero	Olasveengen		MD	Oslo University Hospital, and Institute of Clinical Medicine	University of Oslo, Norwa	Investigator	
